# Septic Arthritis of Neonates: Descriptive Study of a Neonatal Intensive Care Unit Nosocomial Outbreak

**DOI:** 10.7759/cureus.24543

**Published:** 2022-04-27

**Authors:** Nachappa Sivanesan Uthraraj, Srushti Sahukar, Meghana Prakash Hiriyur Prakash, Laya Manasa Sriraam, Siddharth Virani, Gowdar Guruprasad, Jai Relwani

**Affiliations:** 1 Medical Research, Siva Meds Fetal Medicine & Fertility Research (SIMFFER) Foundation, Coimbatore, IND; 2 Trauma and Orthopaedics, East Kent Hospital University Foundation Trust, London, GBR; 3 Neonatology, Bapuji Hospital, Davanagere, IND; 4 Otolaryngology, Siva Meds Fetal Medicine & Fertility Research (SIMFFER) Foundation, Coimbatore, IND; 5 Trauma and Orthopaedics, North and East Hertfordshire NHS Trust, Ashford, GBR; 6 Trauma and Orthopaedics, William Harvey Hospital, Ashford, GBR

**Keywords:** gram negative organism, hip arthrotomy, nosocomial infection, neonates, septic arthritis

## Abstract

Purpose

This is a retrospective descriptive study of a nosocomial outbreak of septic arthritis in a neonatal intensive care unit with a *Pseudomonas* species as the predominant organism. There have been no previous reports of the same. The risk factors for this disease were analysed. The different diagnostic modalities that we used are described and the short-term outcomes are reported after antibiotic therapy and surgery.

Methods

Fourteen patients and 16 joints were included in the study over a three-month period. The risk factors were analysed from the records and included prematurity, birth weight, sex and joint predilection. The causative organisms were also analysed from microbiological profiling. The outcomes after surgery and adjunctive antibiotic therapy were analysed in terms of clinical and laboratory parameters.

Results

*Pseudomonas* *aeruginosa* was found to be the predominant organism in this series. The hip joint was predominantly involved and the majority of the patients were found to be premature. All the neonates affected were found to have low birth weight.

Conclusion

Prematurity and low birth weight were found to have an association with risk for septic arthritis. In our setting of a nosocomial outbreak, a *Pseudomonas* species was more common than other organisms. A treatment regimen of arthrotomy surgery and adjunctive antibiotic therapy was found to be effective in all our patients.

## Introduction

Septic arthritis of neonates is a rare condition. The global incidence is five to 12 cases per 100,000 persons, and among neonates it is 0.12 per 1000 births. In India, it is one in 1500 births [1,2]. This disease is more commonly seen in older children. In neonates, it is most often associated with osteomyelitis and is common in the hip joint. Because of its unique anatomy and blood supply, the metaphysis being intracapsular, the metaphyseal vascular channels penetrate the physeal plate, exposing the hip to the infection [3]. Once the disease sets in, the host immune system, which includes the complement components, phagocytoses the microbes. This mechanism of immunity is poorly developed in neonates [4]. The microbial profile of neonatal septic arthritis is unique as well. Within the literature there is no description of neonatal septic arthritis due to *Pseudomonas* spp. occurring as a nosocomial outbreak in a neonatal intensive care unit (NICU). This is a descriptive study that aims to analyse the sex and joint predilection, risk factors, complications, microbial profile, antibiotic susceptibility, diagnostic modalities, surgical treatment and the effectiveness of the instituitional antimicrobial policy and the short-term outcomes of gram-negative bacteria borne septic arthritis.

## Materials and methods

**Figure 1:**
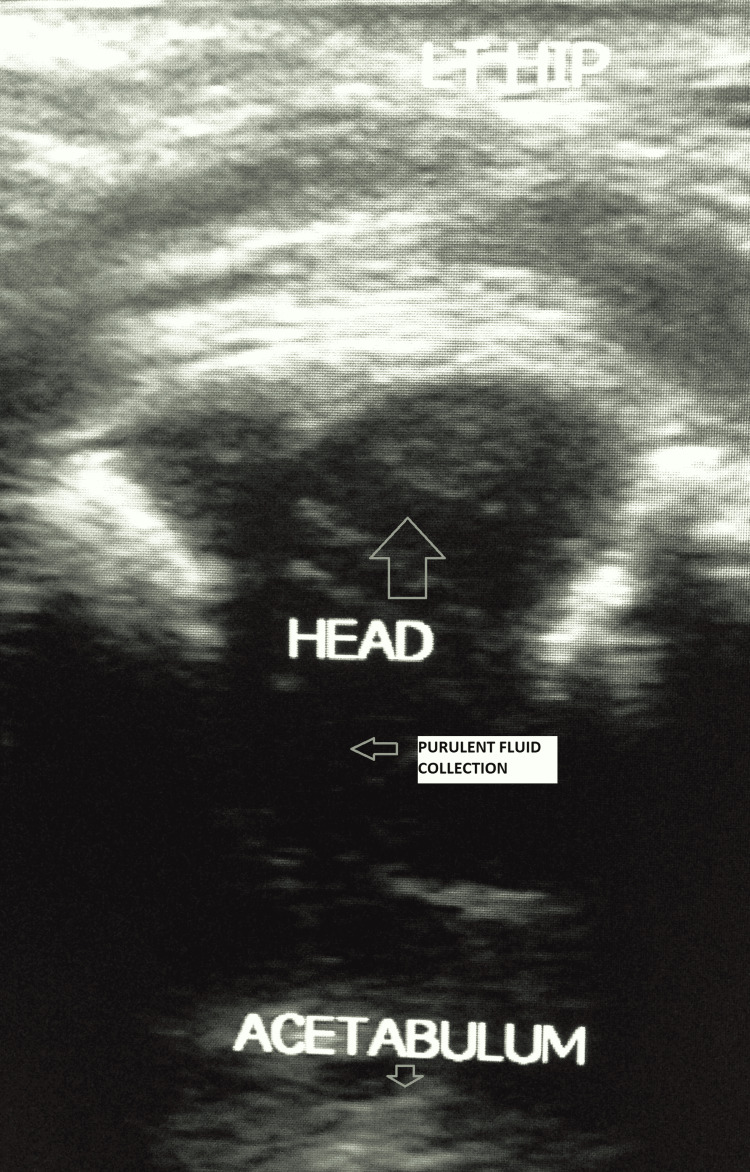
Ultrasound image of the neonatal hip in the axial view showing significant purulent fluid collection in the joint space LT HIP - LEFT HIP

This is a retrospective analysis conducted at a tertiary care institute of a nosocomial neonatal septic arthritis outbreak (Level IV evidence). There were a total of 14 neonates admitted (n=14): six males and eight females, with 16 joints involved. The joints involved were hips (n=14) and knees (n=2). The details were collected with consent from the parent/guardian in accordance with institutional guidelines. The neonates were admitted to a Level III NICU. The period of admission was between March and May 2013, when the outbreak happened. There was one follow-up, three months after discharge. The gender and joint predilection were analysed. The outcomes assessed were risk factors such as gestational age, birth weight, comorbidities, effectiveness of combined surgical and antibiotic therapy, and the organisms cultured. Diagnosis was made by clinical examination, laboratory parameters and ultrasonography. The diagnostic criteria were white blood cell count more than 20000 cells/mm3, C-reactive protein levels of more than 10mg/L, a fluid pocket of more than 7x11 mm on two-dimensional ultrasound scanning, corroborative clinical signs of reduced active joint motion and raised temperature more than 38 degrees Celsius. Ultrasound scan was performed on all the suspected joints by the same sonographer (Figure 1). Fluid pockets in the joint greater than 7x11 mm on the ultrasound scan were deemed significant and included in our case series. The patients were followed up at three months for residual complications. Dosage of intravenous amoxicillin and clavulanic acid was 25mg/kg/dose every 12 hours (dosing is based on the amoxicillin component with clavulanate added in the 5:1 ratio for the total daily dosage) and oral amoxicillin was 20 mg/kg/dose every 12 hours. Amoxicillin and clavulanic acid combination was administered as an intravenous infusion for the first two weeks and subsequently was switched to oral amoxicillin for four weeks as per advice from the institutional microbiology team. Synovial fluid samples from open arthrotomy were cultured on agar plates. The selected antibiotics were continued till the culture and sensitivity reports were available and modified, if required. Surgery was performed on all the 14 patients in our series. The open technique arthrotomy was followed using the anterior approach for hips and medial parapatellar approach for knees by a single general orthopaedic surgeon. A follow-up clinical assessment was done three months later. Informed consent was obtained from the parent (mother) of all the neonates. Ethics committee/institutional review board approval was waived for this study as it was a retrospective case series.

## Results

**Figure 2:**
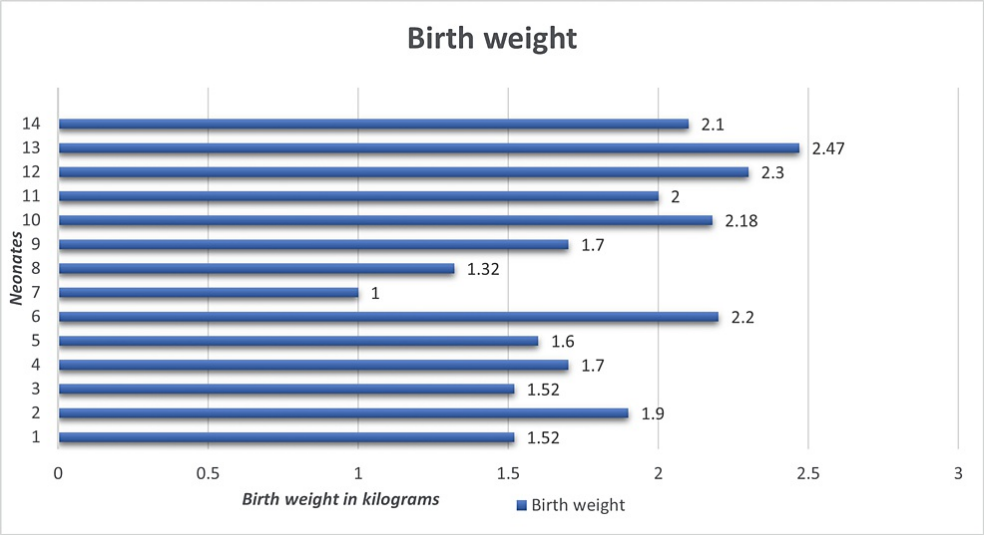
Birth weight of the neonates

The organisms cultured were *Pseudomonas aeruginosa* (56%), *Klebsiella pneumoniae* (25%), and methicillin sensitive *Staphylococcus aureus* (MSSA; 19%) (Table 1). The predominant organism for both preterm and term infants was *Pseudomonas aeruginosa*. The affected neonates were all found to have low birth weight. The average birth weight was 1.8 kg (SD = 0.36) (Figure 2). Five (35.7%) neonates also developed respiratory distress syndrome that required respiratory support in the form of surfactant therapy. Another significant co-morbidity noted was sepsis, which affected five (35.7%) of the neonates in the series. Birth asphyxia (three; 21.4%), hypoxic ischaemic encephalopathy (one; 7.1%), meningitis (one; 7.1%), transient tachypnoea of the newborn (one; 7.1%), and intra-uterine growth retardation (one; 7.1%) were the other co-morbidities (Table 2). Four neonates who did not have any other comorbidities presented on the same day of admission. There was a predilection for females to be affected (males:females = 6:8). The hip joint was affected in 14 neonates (87.5%), followed by the knee in two neonates (12.5%) (Table 3). Multifocal joint involvement (hips and knees) was seen in two patients (12.5%). More than half of the affected babies were preterm births (eight; 61.5%) (Table 4). The average day of admission to the NICU was day eight after birth (SD=6). The C-reactive protein and the white cell count were raised concomitantly for all the patients. The elevated lab parameters declined after the arthrotomies and adjunctive antibiotic therapy which was continued for six weeks (two weeks of intravenous antibiotics and four weeks of oral antibiotics). Three months after discharge, the patients were evaluated on the basis of clinical signs and laboratory parameters. There were no indications of persistent disease. The main cause of the outbreak was found to be inadequate infection control by the staff members and guidelines were formulated to mitigate the risk. This led to no significant outbreaks since then.

**Table 1:** Organisms cultured

Organism	Number (Percentage)
Pseudomonas aeruginosa	9 (56.25%)
Klebsiella sp.	4 (25%)
Methicillin Sensitive Staphylococcus aureus	3 (18.75%)

**Table 2:** Co-morbidities

Co-morbidities	Number (Percentage)
Respiratory distress syndrome	5 (35.7%)
Sepsis	5 (35.7%)
Birth asphyxia	3 (21.4%)
Intrauterine growth retardation	1 (7.1%)
Hypoxaemic ischaemic encephalopathy	1 (7.1%)
Meningitis	1 (7.1%)
Transient tachypnoea of the newborn	1 (7.1%)

**Table 3:** Joint involved

Joint	Number (Percentage)
Hip	14 (87.5%)
Knee	2 (12.5%)

**Table 4:** Prematurity status

Prematurity status	Number (Percentage)
Premature	8 (61.5%)
Term	6 (38.5%)

## Discussion

This case series is the first of its kind to be reported and is unique because of the nosocomial nature of the infections in an NICU setting, over a three-month period and due to the microbial profiling showing *Pseudomonas aeruginosa* as the predominant organism.

Microbial profiling

This series demonstrated that the most dominant microbe was *Pseudomonas aeruginosa*, followed by *Klebsiella pneumoniae* and *Staphylococcus aureus*. There have been no other series or reports with a predominance of *Pseudomonas* spp. or instances of it. Nosocomial infections are mostly due to gram negative organisms [5]. In a retrospective review by Jeyanthi et al. that included six neonates, methicillin resistant *Staphylococcus aureus* was the predominant organism [6]. In the neonatal and the broader paediatric group, *Klebsiella pneumoniae*, *Staphylococcus aureus* and *Escherichia coli* are the commonest organisms. *Staphylococcus aureus* and *Klebsiella pneumoniae* are the most common pathogen in neonates [7-9]. *Salmonella* spp. is implicated in septic arthritis, particularly in developing countries, and sickle cell disease [10]. There are no other similar case series and this probably explains the unusually predominant organism in our series.

Joint involvement

The lower limb and especially the hip is commonly affected [5,8,11-13]. This is congruent with our findings of the hip joint being predominantly involved (87.5%).

Comorbidities

Neonatal resuscitation and comorbidities increase the likelihood of septic arthritis [5]. We found that a majority of the neonates were admitted for other conditions in the NICU (n=8; 56%) and all had effusions requiring drainage.

Sex predilection

The previously reported data is unequivocal in terms of sex predilection with a male predominance [4,9]. Our cohort had a female sex predilection, though not statistically significant.

Prematurity

Prematurity and anaemia are established risk factors for septic arthritis [5,14,15]. This is in line with our findings, with the majority of the neonates being premature.

Birth weight

The case series by Nataraj et al. shows that birth weight has no effect on the development of septic arthritis [5]. Kleigman et al., on the other hand, demonstrated that preterm and low birth weight infants are three to 10 times more likely to develop septic arthritis. These babies tend to be in the NICU for longer periods, increasing manual handling by health care professionals and hence the likelihood of developing disease [15]. All the neonates in our study had low birth weight indicating that low birth weight is a probable risk factor.

Diagnostic modality

In neonatal septic arthritis, the lower limb assumes an attitude of flexion, abduction, and external rotation with swelling [16]. There is decreased active movement (pseudoparalysis). This was evident in all the neonates in our series. Erythrocyte sedimentation rate and C-reactive protein are reliable diagnostic markers in septic arthritis [17]. Real-time polymerase chain reaction is a better tool when available for the detection of gram-positive and gram-negative organisms from synovial fluid samples [9]. Fever, though common in septic arthritis, is difficult to discern in neonates because of physiological neonatal hypothermia [10]. A multicentre review of 45 neonates found positive joint fluid cultures in 85% of the patients [4]. The BACTEC method commonly used for blood cultures is superior to the conventional agar plate method for synovial fluid samples as well [12]. The diagnosis was made by an initial assessment of the vitals, physical examination, and blood tests. Joint ultrasound is indicated when osteoarticular infection in neonates is suspected [18]. Neonates with suspected septic arthritis as determined by high temperature, pseudoparalysis, elevated white cell count, and C-reactive protein levels underwent ultrasound examination followed by an open arthrotomy with synovial and purulent fluid culture in our study.

Antimicrobial treatment

Cefazolin is the empirical antibiotic of choice in neonates. However the choice of antibiotic therapy is by local protocol. There is no clear consensus on the duration of antimicrobial treatment or mode of delivery for this condition [12]. The concentration of the antibiotic in the synovial fluid equals the serum concentration one hour after infusion, making intravenous infusion a very effective delivery mechanism for acute infection [13]. A shortened course of IV therapy of one week was found to be just as effective as the conventional regimen of intravenous and oral therapy for six weeks by Jagodzinski et al., but this applies mainly to the paediatric population and can differ with neonates [19]. We followed the institutional protocol and started all the babies on empirical amoxiclav and amikacin as an intravenous infusion for two weeks and oral amoxiclav for four weeks. After isolating the organism and testing for susceptibility, there was no need for us to change antibiotics, as the isolates were sensitive to either or both. All neonates were discharged from the NICU at varying periods with no recurrence three months after discharge.

Surgical treatment

Avascular necrosis can develop in the hip if septic arthritis is neglected, or if the drainage is delayed [7]. Kabak et al. found that 86% of the neonates treated with arthrotomy and drainage improved without any sequelae [2]. Surgery should always be performed as an adjunct to antibiotic therapy [20]. There is no difference in the outcomes between surgical and non-surgical treatment two weeks after the onset of symptoms [7]. Hip and shoulder septic arthritis should be considered emergency conditions necessitating an arthrotomy procedure to preserve the joint [20]. Arthroscopic lavage and debridement of the neonatal septic hip have the advantages of less “procedures required per patient” (1.125) and shorter hospital stays when compared to the arthrotomy group. The disadvantage is the unavailability of appropriate trocar cannulas and using substitutes puts the neural structures around the hip joint at risk [21]. Ilharreborde et al. were able to establish that a maximum delay period of four days before starting surgical or conservative treatment ensures that the joints heal without sequelae [18]. The anterior approach to the hip is used for arthrotomy in infants and children with septic arthritis [22]. We used the same technique for the arthrotomies. In our center, we were able to start antibiotics immediately and surgery was carried out for the chosen joints within 48 hours from the diagnosis. This gave us very good short-term outcomes.

Sequelae

Delays in diagnosis and treatment of septic arthritis are the most common causes of sequelae [23]. Concomitant osteomyelitis at any age less than one can predispose to the sequelae of septic arthritis [20]. Ruben et al. have shown that all the joints involved in their study had osteomyelitis before or after [4]. There has been a report of septic arthritis of the hip joint, with a subperiosteal abscess in the neck of the femur which resolved, producing an intraosseous abscess in the medial condyle of the femur four years later by the same strain of the extended-spectrum beta-lactamase producing *Klebsiella pneumoniae* [24]. Septic arthritis in the neonate can result in growth discrepancy due to physeal injury, which manifests as an angular or length deformity [14]. There were no cases with concomitant osteomyelitis in our series which would have increased the chances of sequelae when followed up at three months.

Limitations

Though this case series is the first of its kind, there are some limitations. We could not follow up with patients beyond three months at that time because the healthcare setup in India is in most cases independent of insurance and it may be described as arbitrary. This has harmed and continues to harm physician-patient continuity, care continuity, and needless to say research and academic follow-up. This is compounded by a lack of awareness among patient families who fail to attend follow-up appointments and the fact that digital India and overall connectivity was still in its infancy when this study was set up. This is also a window into how technology (or lack of it) can affect outcomes. A more extended follow-up would have helped us report sequelae or long-term complications if any. There was no control group for comparison.

## Conclusions

From this series we were able to conclude that *Pseudomonas aeruginosa*, though not previously reported, can be a case of nosocomial septic arthritis in neonates. Prematurity and low birth weight had an influence on the development of septic arthritis. Amoxiclav with amikacin as an intravenous infusion for two weeks and amoxiclav orally for four weeks with adjunct open arthrotomies within 48 hours are sufficient for managing infections due to *Pseudomonas aeruginosa*, *Klebsiella pneumoniae* and *Staphylococcus aureus* without sequelae in the short term.
